# Case Report: Telitacicept in the treatment of systemic lupus erythematosus complicated by acquired hemophilia A

**DOI:** 10.3389/fimmu.2026.1733996

**Published:** 2026-03-12

**Authors:** Xiaoyang Liu, Wenqian Li

**Affiliations:** Department of Hematology and Rheumatology, Qinghai Provincial People’s Hospital, Xining, China

**Keywords:** acquired hemophilia A, BLyS/APRIL dual-target inhibitor, factor VIII inhibitor, systemic lupus erythematosus, telitacicept

## Abstract

**Background:**

Systemic lupus erythematosus (SLE) is a multisystem autoimmune disease. Acquired hemophilia A (AHA) is a rare acquired bleeding disorder characterized by the presence of autoantibodies against coagulation factor VIII (FVIII) in the circulation, leading to reduced FVIII activity. Cases of SLE complicated by AHA are scarce and challenging to treat. Conventional immunosuppressants are often associated with inadequate efficacy or adverse effects. Telitacicept, a dual-target biologic agent against B-cell activating factor (BAFF) and a proliferation-inducing ligand (APRIL), offers a novel therapeutic approach for such refractory comorbidities.

**Case introduction:**

This report describes a 52-year-old female with a 10-year history of SLE, previously diagnosed with concomitant Sjögren’s syndrome and antiphospholipid antibody syndrome currently seronegative for respective antibodies following prolonged immunosuppression. Her disease was recurrent despite long-term corticosteroid and immunosuppressive therapy. She was later diagnosed with SLE-associated AHA due to fever, epistaxis, and coagulation abnormalities. Subsequently, half-dose telitacicept (80mg/week, adjusted due to financial constraints) combined with corticosteroids was administered. The patient experienced reduced bleeding symptoms, decreased FVIII antibody levels, no recurrent bleeding during follow-up, and stable disease.

**Conclusion:**

Half-dose telitacicept combined with corticosteroids effectively controlled the disease in this patient with SLE-associated AHA, improving bleeding symptoms and coagulation function while allowing corticosteroid dose reduction. This provides a feasible treatment option for such patients with limitations to conventional therapy or financial constraints. However, efficacy differences between different dosages and its long-term safety still require further verification.

## Introduction

Systemic lupus erythematosus (SLE) is a systemic autoimmune disease characterized by involvement of multiple systems and organs, alternating relapse and remission, and the presence of numerous autoantibodies. Without timely treatment, it can cause irreversible damage to affected organs, potentially leading to death (1). Acquired hemophilia A (AHA) is an acquired bleeding disorder resulting from the appearance of autoantibodies targeting endogenous FVIII, leading to decreased FVIII activity (FVIII:C). Its incidence is low, and approximately 50% of cases are secondary to conditions like autoimmune diseases (e.g., SLE), malignancies, drugs, or infections (2). SLE complicated by AHA is extremely rare. These patients not only face challenges from active SLE but also have a high bleeding risk due to coagulation abnormalities, significantly increasing treatment difficulty. Telitacicept, as the first dual-target inhibitor of BLyS and APRIL, works by specifically binding to B lymphocyte stimulator (BLyS) and a proliferation-inducing ligand (APRIL), inhibiting the activation, proliferation, and differentiation of B lymphocytes, and reducing plasma cell generation and autoantibody secretion. It has shown efficacy in refractory autoimmune diseases (3). This article reports a case of telitacicept treating SLE-associated AHA, aiming to provide new insights for managing this rare complication.

## Literature review

SLE complicated by AHA is exceedingly rare in clinical practice. To contextualize this case within the existing literature, we systematically searched the PubMed, Embase, and China National Knowledge Infrastructure (CNKI) databases up to January 24, 2026, using the keywords “systemic lupus erythematosus,” “acquired hemophilia A,” and “factor VIII inhibitor.” This search identified 9 eligible Chinese and English case reports or case series, the key features of which are summarized in **Table 1**.

**Table 1:** Summary of previously reported cases of SLE-complicated AHA.

Author (Year)	Sex	Age (yr)	Diagnostic sequence	Major clinical manifestations	Treatment regimen	Outcome	Remarks
Trotta et al. (1999) (4)	F	19	SLE (4 years) → AHA	Sudden metrorrhagia requiring transfusion, extensive ecchymosis	Initial: Plasmapheresis + IVIG + Prednisolone + FVIII concentrate (ineffective); Later added Cyclophosphamide (pulse 750 mg/m² + oral 100 mg/d)	Long-term remission (FVIII normalized, no recurrence at 4-year follow-up)	Associated with positive lupus anticoagulant (LAC)
Aziz et al. (2016) (5)	M	70	AHA presentation → SLE diagnosis	Generalized ecchymosis, massive right hand hematoma, anemia (Hb 6 g/dL), arthralgia	Prednisolone (1 mg/kg/d) + Fresh frozen plasma	Hematoma resolved and ecchymosis absorbed after 1 week; discharged for follow-up	Male patient, concomitant autoimmune hemolytic anemia (Coombs positive)
Erdem (2012) (6)	M	22	Quiescent SLE (3 years) → Multiple factor inhibitors	Epistaxis, spontaneous ecchymoses over elbows, knees, and ankles	Monotherapy Oral Prednisolone (1 mg/kg/d)	Improved at 3 months; complete normalization of coagulation tests at 6 months	Male patient with inhibitors against FII, FVIII, FIX, FX, and vWF; LAC positive
Akahoshi et al. (2008) (7)	F	38	SLE (1 year) → AHA	Spontaneous ecchymoses (elbow, ankle), hematuria, subconjunctival hemorrhage, thoracic zoster	Methylprednisolone pulse (1 g/d × 3d) → Prednisolone 60 mg/d + Cyclosporine (200 mg/d) + Cyclophosphamide pulse (900 mg)	At 10 weeks: FVIII 94%, inhibitor 1 BU/mL; no recurrence at 2-year follow-up	Extremely high inhibitor titer (1,320 BU/mL); active SLE (SLEDAI = 8)
Shen et al. (2020) (8)	F	51	SLE → AHA (2 months after SLE diagnosis)	Limb and abdominal ecchymosis, hematuria, left lower extremity hematoma, bilateral leg edema, pericardial effusion, ascites	Methylprednisolone 80 mg/d + Cyclophosphamide 200 mg/week + Recombinant FVIII (ADVATE) + Plasmapheresis + Fresh plasma	APTT and FVIII improved after 17 days; discharged with inhibitor 0.62 BU/mL; complete remission on follow-up	Concomitant thrombocytopenia (37 × 10^9^/L), active SLE
Ishikawa et al. (2001) (9)	F	24	Quiescent SLE (5 years) → AHA	Persistent bleeding for 3 weeks after tooth extraction, nasal bleeding, subcutaneous hemorrhage	Prothrombin complex concentrate (PCC) + Activated PCC (aPCC) for hemostasis, followed by Prednisolone + Cyclophosphamide (100 mg/d)	At discharge: inhibitor 0.95 BU/mL, FVIII activity 30%; long-term follow-up without recurrence	SLE in clinical remission; possible association with cefdinir allergy
You et al. (2017) (10)	F	80	AHA and SLE simultaneous diagnosis	Recurrent oral bleeding, bruising of extremities and hips, gross hematuria, photosensitivity, painless oral ulcer	Monotherapy Oral Prednisolone (titrated from 5 mg to 20 mg)	After 4 months: bleeding tendency resolved, APTT decreased from 68.5s to 47.3s, SLEDAI decreased from 6 to 2	Elderly patient (80 years); responded well to steroids alone without cyclophosphamide
Rezaieyazdi et al. (2011) (11)	F	37	Autoimmune hepatitis (5 years) → SLE + AHA	Fatigue, abdominal pain, hematuria, large left flank ecchymosis, leukopenia	Fresh frozen plasma (10 mL/kg) + Methylprednisolone pulse (1000 mg/d × 3d) → Prednisolone 60 mg/d + Cyclophosphamide pulse	At 8 weeks: FVIII activity 135%, inhibitor negative; no hemorrhagic recurrence at 1.5-year follow-up	Extremely high inhibitor titer (>200 BU/mL); active SLE (SLEDAI = 11)
Ye et al. (2023) (12)	F	29	SLE (10 years) → AHA	Massive intra-abdominal hemorrhage (2500 mL), pelvic hematomas, incisional oozing, vaginal bleeding, severe anemia (Hb 22–45 g/L)	Methylprednisolone pulse (1000 mg × 3d) + IVIG (20 g × 3d) + Cyclophosphamide (0.4 g/week) + Blood products + Vascular embolization + Rituximab (100 mg/week × 6)	Bleeding arrested at 7 weeks; APTT normalized (26.7s); inhibitor 0.59 BU/mL (negative); 5-year follow-up stable	High-titer inhibitor (31.2 BU/mL); poor response to steroids + CTX, then switched to Rituximab

SLE, Systemic lupus erythematosus; AHA, Acquired hemophilia A; F, Female; M, Male; FVIII, Factor VIII; BU, Bethesda units; LAC, Lupus anticoagulant; IVIG, Intravenous immunoglobulin; CTX, Cyclophosphamide; RTX, Rituximab; SLEDAI, SLE Disease Activity Index; APTT, Activated partial thromboplastin time; PCC, Prothrombin complex concentrate; aPCC, Activated prothrombin complex concentrate; Hb, Hemoglobin.

## Case presentation

**Figure 1:**
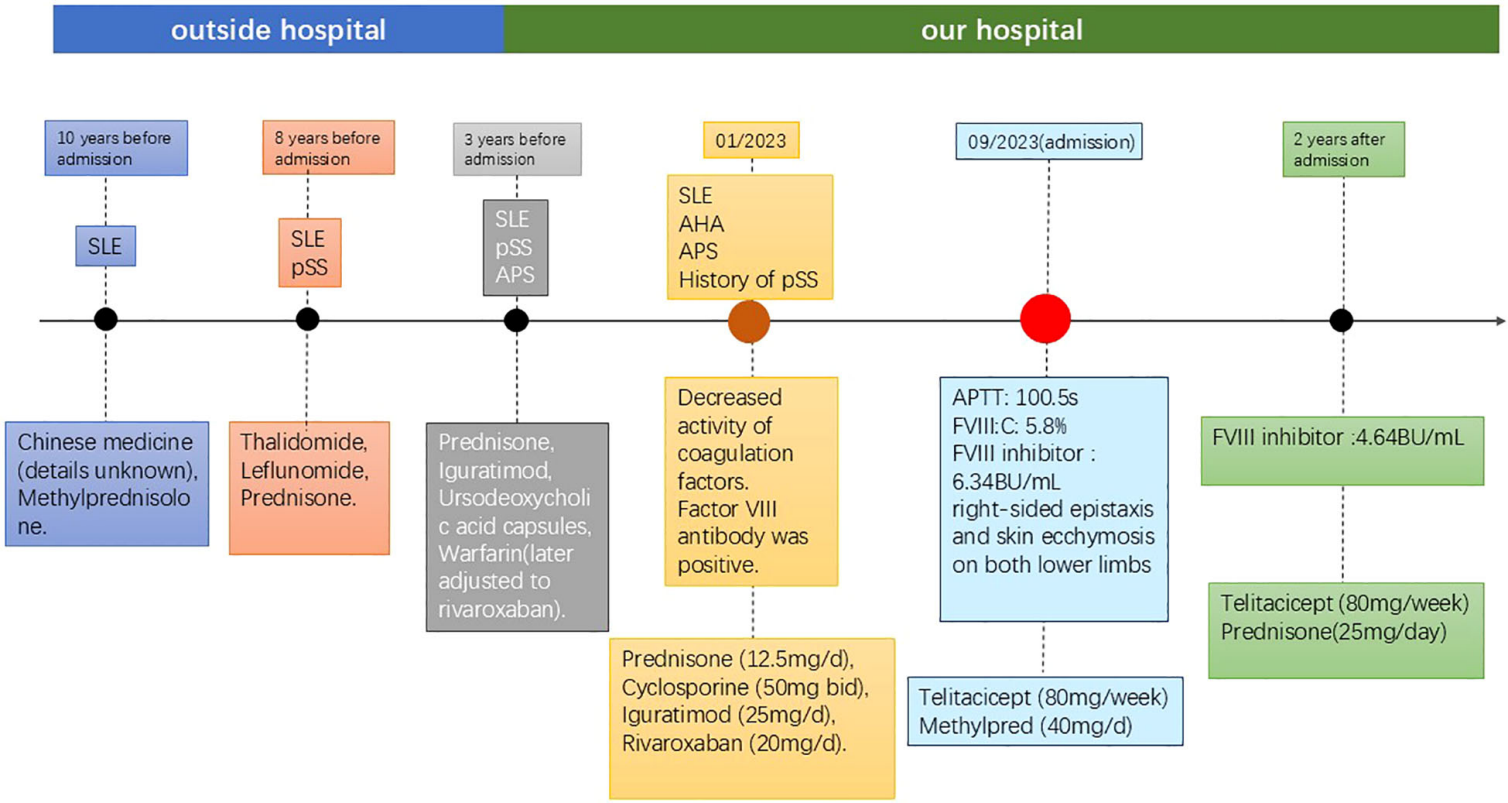
Timeline of clinical course and therapeutic interventions. Key events are shown relative to the time of admission for AHA management (index date). SLE, systemic lupus erythematosus; pSS, Sjögren’s syndrome; AHA, acquired hemophilia A.

A 52-year-old female was admitted due to recurrent joint swelling and pain, dry mouth and eyes with fever for over 10 years, aggravated with skin ecchymosis and epistaxis for 1 week. (**Figure 1**) Over 10 years ago (around 2013), the patient experienced unexplained generalized pain, accompanied by dry mouth, dry eyes, and recurrent low-grade fever (37.5–38.6 °C). She was diagnosed with SLE at Qinghai Provincial Hospital of Traditional Chinese Medicine and treated with traditional Chinese medicine (details unknown) and methylprednisolone (starting at 8 tablets/day, specific dose unknown), with partial symptom relief. In 2015, following an episode of fever (temperature not recorded), the patient was diagnosed with Sjögren’s syndrome at Xining First People’s Hospital. The diagnosis was established based on a lip biopsy showing focal lymphocytic sialadenitis, accompanied by clinical manifestations consistent with keratoconjunctivitis sicca, meeting the 2012 ACR classification criteria. She was initially treated with thalidomide and leflunomide, but disease relapse was prone to occur when corticosteroids were tapered to methylprednisolone 8 mg/day. Between 2020 and 2021, she was additionally diagnosed with antiphospholipid antibody syndrome (APS). This diagnosis was based on persistent positivity for lupus anticoagulant, confirmed on two occasions at least 12 weeks apart, in accordance with the 2006 Sydney criteria, although no thrombotic events were documented. This diagnosis subsequently required long-term anticoagulation therapy. In February 2020, she was admitted to our department due to disease activity, and her treatment regimen was adjusted to prednisone, iguratimod, ursodeoxycholic acid capsules, and warfarin. In November 2021, she was re-admitted for fever and granulocytopenia; she received antiviral therapy during this hospitalization, and her anticoagulant was switched from warfarin to rivaroxaban (20 mg/day) while continuing the rest of the previous treatment regimen. At the time of admission for AHA management in September 2023, repeat testing for anti-SSA/Ro, anti-SSB/La, anticardiolipin antibodies (IgG/IgM), and anti-β2-glycoprotein I antibodies was negative, while lupus anticoagulant remained positive. Although the archived laboratory records from the external hospital documenting the original antibody status are unavailable, this current serological profile—negativity for extractable nuclear antigens and phospholipid antibodies—suggests that long-term immunosuppressive therapy over the preceding years may have suppressed the production of these specific autoantibodies, while the lupus anticoagulant activity persisted. In January 2023, after catching a cold, she developed a fever up to 39.5 °C, with dry cough, epistaxis, and abnormal coagulation (PT 16.1s ↑, APTT 92.3s ↑). Coagulation factor tests revealed reduced activity of factors V, VII, VIII, IX, X, XI, XII, notably factors VIII, IX, XI, XII. Factor VIII antibody was positive. The diagnosis was consistent with SLE-associated AHA. Vitamin K was administered. Rituximab was recommended for inhibitor eradication, but the patient refused due to financial reasons. Post-discharge, she regularly took prednisone (12.5mg/d), cyclosporine (50mg bid), iguratimod (25mg/d), and rivaroxaban (20mg/d).

In September 2023, the patient experienced right-sided epistaxis and skin ecchymosis on both lower limbs. Repeat coagulation tests showed: PT 14.3 sec ↑, APTT 100.5 sec ↑. She was admitted to our department with “SLE, abnormal coagulation function”. On admission, she had a mild anemic appearance, erosion of the right nasal mucosa with minimal oozing, and scattered non-blanchable ecchymosis on both lower limbs. Cardiopulmonary and abdominal examinations were unremarkable; joints showed no swelling or tenderness. Admission coagulation: PT 14.5s (ref 10-14s), APTT 100.5s (ref 23.3-32.5s), APTT ratio 3.6 (ref 0.73-1.27). Coagulation factors: FVIII:C 5.8% ↓ (ref 50-150), FIX:C 1.1% ↓ (ref 50-150). CBC: WBC 1.32x10^9^/L ↓, Hb 116 g/L ↓, PLT 114x10^9^/L, manual neutrophil count 0.25x10^9^/L ↓↓, reticulocyte percentage 3.02%. Urinalysis revealed microscopic hematuria with 9.76 red blood cells per high-power field (RBC/HPF) (occult blood 2+, RBC 54.2/μL), while protein was negative. Inflammatory markers: ESR 90 mm/h, CRP 16.296 mg/L ↑, IL-6 10.5 pg/mL ↑. Biochemistry: Total protein 90.8 g/L ↑, Albumin 33.9 g/L ↓, Globulin 56.9 g/L ↑, β2-microglobulin 4.36 μg/mL ↑, K + 3.46 mmol/L ↓, Na+ 133 mmol/L ↓. Special proteins: Complement C4 (C4) 9 mg/dL ↓, IgG 3098 mg/dL ↑, IgM 891 mg/dL ↑. T-cell subsets/Lymphocyte subsets: Total T cells 89.26% ↑, CD4/CD8 ratio 1.3 ↓, NK cells 4.56% ↓, B cells 5.0% (at lower limit of normal). Autoantibodies: ANA homogeneous pattern positive 1:3200, anti-dsDNA positive 1:320, anti-nRNP +++, anti-Sm ++, anti-histone ++, anti-mitochondrial antibody M2 (AMA-M2) +++. Lupus anticoagulant testing: LA screening test 122.3 sec ↑, LA confirmation test 49.1 sec ↑. Notably, despite the historical diagnoses, repeat testing revealed negative anti-SSA/Ro and anti-SSB/La antibodies, as well as negative anticardiolipin antibodies (IgG/IgM) and anti-β2-glycoprotein I antibodies. Lupus anticoagulant was positive. This seronegative conversion was attributed to sustained immunosuppressive therapy over the preceding years.

Based on the 2019 EULAR/ACR SLE classification criteria and AHA diagnostic guidelines, the diagnosis was confirmed as: Systemic Lupus Erythematosus (active phase) complicated by Acquired Hemophilia A (Factor VIII inhibitor positive).

The patient had a 10-year history of SLE and had been admitted multiple times previously. This admission was for right epistaxis and lower limb ecchymosis. SLE disease activity was assessed using the SLE Disease Activity Index 2000 (SLEDAI-2K). The patient’s total score was 11, attributed to microscopic hematuria (4 points, with 9.76 RBC/HPF), mucosal ulcers (2 points, nasal mucosal erosion), leukopenia (1 point, WBC 1.32 × 10^9^/L), low complement C4 (2 points, 9 mg/dL), and elevated anti-dsDNA antibodies (2 points, 1:320). This score is consistent with moderate disease activity. The presentation was further complicated by AHA (FVIII inhibitor 6.34 BU/mL) with a clear bleeding tendency. Previous treatments with leflunomide and iguratimod had resulted in granulocytopenia, and she was sensitive to corticosteroid reduction (relapse at ≤8mg/day), consistent with poor response or adverse effects to conventional immunosuppressants. Treatment focus was controlling lupus activity, suppressing FVIII/FIX inhibitors, and managing bleeding. she had previously refused rituximab due to cost. Considering active SLE and AHA, where conventional immunosuppressants might increase bleeding risk, telitacicept, a dual-target biologic inhibiting BLyS and APRIL to suppress B-cell activation/proliferation, was considered due to its efficacy in SLE and favorable safety. After discussion with the family, telitacicept combined with corticosteroids was initiated.

Treatment Plan: Telitacicept 80mg/subcutaneous injection once weekly (recommended starting dose 160mg; half-dose used due to financial constraints); Methylprednisolone 40mg intravenous drip once daily. Concurrently, cryoprecipitate and plasma were transfused to improve coagulation and provide supportive care. Weekly monitoring included CBC, coagulation, inflammatory markers; bi-weekly monitoring of liver/kidney function, autoantibodies (dsDNA, ANA); monthly assessment of SLE activity and bleeding symptoms.

Two weeks after treatment, no new ecchymosis developed, the right epistaxis resolved, APTT decreased to 86.5s, and FVIII:C was 5.6%. After 3 months, clinical symptoms largely resolved: APTT 79.8s, ESR 26 mm/h, CBC: WBC 4.23 x 10^9^/L ↓, Hb 135 g/L ↓, PLT 108 x 10^9^/L. Corticosteroids were tapered to 25mg/day; telitacicept continued at 80mg/week. After 6 months, APTT decreased to 68.3s, CBC improved (WBC 3.69 x 10^9^/L, Hb 137 g/L, PLT 128 x 10^9^/L); anti-dsDNA antibody titers decreased from 1:320 to 1:100, and complement C3 and C4 levels normalized (C3 120.6 mg/dL, C4 15mg/dL). Corticosteroids reduced to 20mg/day; telitacicept maintained. After 2 years, FVIII inhibitor titer decreased to 4.64 BU. Corticosteroids were gradually tapered to 10mg orally daily; telitacicept continued at 80mg/week (**Table 2**).

**Table 2:** Serial laboratory findings in the current patient.

Timepoint	APTT (s)	FVIII:C (%)	FVIII Inhibitor (BU/mL)	Anti-dsDNA	C3 (mg/dL)	C4 (mg/dL)	Hemoglobin (g/L)	Platelet (×10^9^/L)	Treatment Modifications	Bleeding Status
Baseline (Admission)	100.5	5.8	6.34	1:320 positive	100	9	116	114	Methylprednisolone 40mg IV, Telitacicept 80mg SC	Epistaxis, ecchymosis
Week 2	86.5	5.6	—	—	—	—	123	123	Continue same	No new ecchymosis
Month 3	79.8	—	—				135	108	Prednisone tapered to 25mg/d	Symptoms largely resolved
Month 6	68.3	—	—	1:100 positive	110.3	14	137	128	Prednisone 20mg/d	No bleeding, stable condition
Year 2	64.4	7.8	4.64		120.6	15	132	107	Prednisone 10mg/d	No fever, bleeding, or joint pain

Throughout the 24-month follow-up period, the patient experienced no injection site reactions (erythema, pain, pruritus, or induration), infections (including upper respiratory or urinary tract infections), or other drug-related adverse events while receiving weekly subcutaneous telitacicept at 80 mg. The patient attended regular follow-ups without fever, bleeding, or arthralgia. Repeated tests showed normalizing trends and reduced inhibitor levels. Quality of life significantly improved, enabling normal work and daily activities. Long-term follow-up further verified the efficacy and stability of telitacicept treatment.

APTT, activated partial thromboplastin time; FVIII:C, factor VIII activity; BU, Bethesda units; SLEDAI-2K, Systemic Lupus Erythematosus Disease Activity Index 2000; SC, subcutaneous; IV, intravenous.

## Discussion

SLE is an autoimmune disease affecting multiple systems. Hematological involvement is common, including anemia, leukopenia, and thrombocytopenia, related to autoantibody-mediated destruction and bone marrow suppression. However, SLE-associated AHA is rare. AHA involves autoantibodies (inhibitors) against coagulation factor s, causing coagulopathy and bleeding. Its pathogenesis may relate to the complex immune dysregulation in SLE, where autoantibodies might cross-react with coagulation factor s. AHA carries high bleeding risk and is difficult to treat. Traditional regimens include immunosuppressants and factor replacement, but some patients respond poorly or experience adverse effects.

A systematic review of previously reported SLE-associated AHA cases reveals consistent patterns in patient demographics and therapeutic challenges. As summarized in **Table 1**, the majority of reported patients were young to middle-aged females (median age 37 years, range 19–80), with a substantial proportion receiving cyclophosphamide-based regimens as first-line therapy and prednisone monotherapy effective in some cases; rituximab was reserved for refractory cases (4–12). These conventional approaches, while effective in 60–70% of cases, typically require 2–12 weeks to achieve inhibitor eradication and carry significant risks of myelosuppression, infection, and malignancy. In contrast, the present case exhibited a distinctive triple autoimmune phenotype (SLE complicated by Sjögren’s syndrome and APS, with documented positive anti-SSA/SSB antibodies and antiphospholipid antibodies) and prior intolerance to conventional immunosuppressants, highlighting the need for alternative therapeutic strategies that target B-cell hyperactivity without overlapping toxicities.

This case involved SLE with AHA, positive for FVIII inhibitor and significant bleeding, core mechanism being autoantibody production. BLyS and APRIL levels are elevated in active SLE, correlating positively with ESR, CRP, anti-dsDNA, and negatively with C3/C4 (13). B-cell maturation antigen (BCMA), expressed during terminal B-cell differentiation into plasma cells, is a key receptor for BLyS/APRIL. APRIL binding to BCMA activates pathways like NF-κB, upregulating anti-apoptotic proteins (e.g., Bcl-2, Mcl-1), sustaining long-lived plasma cells and antibody secretion (14), with expression levels correlating with disease activity (15).This suggests that hyperactivity of the BLyS/APRIL pathway plays a key role in the production of various autoantibodies, including coagulation factor inhibitors.

Telitacicept, a recombinant TACI-Fc fusion protein, is a dual-target biologic blocking both BLyS and APRIL, inhibiting mature B-cell to plasma cell differentiation and reducing antibody secretion (16), showing efficacy in autoimmune diseases. A phase IIb trial (n=249) showed SRI-4 response rate of 77.8% at 48 weeks with 160 mg/week vs 50% for placebo, with reduced anti-dsDNA and increased complement (17). A phase III study (NCT04082416) confirmed SRI-4 of 82.6% at 52 weeks, with effects evident from week 4 (18). Real-world studies confirm clinical remission in SLE patients with nephritis or hematological abnormalities without increased infection risk (19).

Pharmacokinetic studies demonstrate that subcutaneous telitacicept 160 mg achieves peak plasma concentration within 1–2 days and reaches steady state after 7 weeks, with linear pharmacokinetics observed across single doses of 80–240 mg (20–22). This linear relationship supports dose-proportional exposure, suggesting that the 80 mg/week regimen used in this case (reduced from the standard 160 mg/week due to financial constraints) could maintain therapeutically relevant drug levels. This case demonstrates that prolonged treatment with reduced-dose telitacicept (80 mg/week) not only achieved marked clinical benefits—including significant improvement in bleeding symptoms, progressive reduction in FVIII inhibitor titers, and avoidance of recurrent hemorrhage or hospitalization throughout the 24-month follow-up—but also maintained an excellent safety profile without observed treatment-emergent adverse events. This favorable efficacy-safety balance, consistent with data from pivotal SLE trials (8, 10), supports the potential for dose individualization based on patient tolerability and economic factors. While this case demonstrates efficacy at the reduced dose, the authors hypothesize that the standard 160 mg/week regimen might achieve more profound BLyS/APRIL pathway inhibition, potentially accelerating FVIII inhibitor clearance and shortening the time to bleeding control by further suppressing plasma cell survival and autoantibody production. This hypothesis, however, requires validation through prospective comparative studies, and dosing should be individualized based on therapeutic response, tolerability, and economic considerations.

Although no prospective studies exist for telitacicept specifically in AHA, case reports in antibody-mediated rare complications like MOG antibody disease and NF155+ autoimmune nodopathy show specific autoantibody titers decrease significantly with treatment alongside clinical improvement (23, 24). Thus, telitacicept, by inhibiting BLyS/APRIL, may reduce coagulation factor inhibitor production at the source, potentially becoming an effective intervention for SLE-AHA, requiring larger sample validation.

## Conclusion

This case demonstrates that telitacicept may be an effective treatment for SLE patients with secondary AHA who have an inadequate response or intolerance to conventional immunosuppressants. By dual inhibition of the BLyS/APRIL pathway, telitacicept reduces the production of coagulation factor inhibitors at the source, thereby relieving bleeding symptoms and facilitating corticosteroid tapering. Pharmacokinetic data support effective concentration maintenance with 80mg/week (11–13), and 2-year follow-up showed no infections or other adverse effects, consistent with reported long-term safety (8, 10). As no prospective studies exist for telitacicept in AHA, subsequent multicenter registry studies are recommended to further validate its efficacy and optimal dosing in this population.

## Data Availability

The raw data supporting the conclusions of this article will be made available by the authors, without undue reservation.

## References

[1] National Clinical Research Center for Dermatologic and Immunologic Diseases Chinese SLE Treatment and Research Group Rheumatology Branch of Chinese Medical Association . Chinese guideline for the diagnosis and treatment of systemic lupus erythematosus (2025 edition). National Medical Journal of China. (2025) 105:1879–906. 10.3760/cma.j.cn112137-20250228-00479 40495587

[2] Thrombosis and Hemostasis Group, Chinese Society of Hematology, Chinese Medical Association Hemophilia Cooperation Group of China . Chinese guidelines on the diagnosis and treatment of acquired hemophilia A (2021). Chinese Journal of Hematology. (2021) 42:793–9. doi: 10.3760/cma.j.issn.0253-2727.2021.10.001, PMID: 34788917 PMC8607020

[3] Samy E Wax S Huard B Hess H Schneider P . Targeting BAFF and April in systemic lupus erythematosus and other antibody-associated diseases. Int Rev Immunol. (2017) 36:3–19. doi: 10.1080/08830185.2016.1276903, PMID: 28215100

[4] Trotta F Bajocchi G La Corte R Moratelli S Sun L . Long-lasting remission and successful treatment of acquired factor VIII inhibitors using cyclophosphamide in a patient with systemic lupus erythematosus. Rheumatology. (1999) 38:1007–9. doi: 10.1093/rheumatology/38.10.1007, PMID: 10534554

[5] Aziz MA Ahmed F Islam S Islam S Khan R Begum M . Auto antibody mediated acquired haemophilia: A case report. Bangabandhu Sheikh Mujib Med Univ J. (2016). 9:44–47 doi: 10.3329/bsmmuj.v9i1.28943

[6] Erdem O . Acquired inhibitors to coagulation factors in a Male patient with systemic lupus erythematosus: a case report and review of the literature. Int J Hematol Oncol. (2012). 22:120–124 doi: 10.4999/uhod.09155

[7] Akahoshi M Aizawa K Nagano S Inoue H Sadanaga A Arinobu Y . Acquired hemophilia in a patient with systemic lupus erythematosus: a case report and literature review. Mod Rheumatol. (2008) 18:511–5. doi: 10.1007/s10165-008-0084-6, PMID: 18551353

[8] Shen P Li J Tu S Chen G Chen C . Acquired hemophilia a in a woman with systemic lupus erythematosus. Med (Baltimore). (2020) 99:e22926 doi: 10.1097/md.0000000000022926, PMID: 33120848 PMC7581163

[9] Ishikawa T Tsukamoto N Suto M Inoue H Sadanaga A Arinobu Y . Acquired hemophilia a in a patient with systemic lupus erythematosus. Intern Med. (2001). 129:316–9 doi: 10.2169/internalmedicine.40.541, PMID: 11446683

[10] You J Kim H Park JS Chang MH Lee CH . Acquired hemophilia a combined with systemic lupus erythematosus: a case report and literature review. J Rheum Dis. (2017), 309–12 doi: 10.4078/jrd.2017.24.5.309, PMID: 37476524

[11] Rezaieyazdi Z Sharifi-Doloui D Hashemzadeh K Shirdel A Mansouritorghabeh H . Acquired haemophilia A in a woman with autoimmune hepatitis and systemic lupus erythematosus: review of literature. Blood Coagul Fibrinolysis: Int J Haemost Thromb. (2011) 22:738–41. doi: 10.1097/MBC.0b013e32834a5c8e, PMID: 21885954

[12] Ye M Deng RY Shen FC Hou ZF Lin L . Systemic lupus erythematosus complicated with acquired hemophilia A: A case report and literature review. Journal of Central South University (Medical Sciences). (2023) 48:789–94. doi: 10.11817/j.issn.1672-7347.2023.220440, PMID: 37539582 PMC10930402

[13] Shater H Fawzy M Farid A El-Amir A Fouad S Madbouly N . B-cell activating factor and a proliferation-inducing ligand in relation to intima-media thickness as biomarkers of premature atherosclerosis in systemic lupus erythematosus patients. Am J Med Sci. (2022) 364:646–54. doi: 10.1016/j.amjms.2022.05.008, PMID: 35580639

[14] Cho SF Lin L Xing L Li Y Yu T Anderson K . BCMA-targeting therapy: driving a new era of immunotherapy in multiple myeloma. Cancers. (2020) 12:1473. doi: 10.3390/cancers12061473, PMID: 32516895 PMC7352710

[15] Sanchez E Smith EJ Yashar MA Patil S Li M Porter A . The role of B-cell maturation antigen in the biology and management of, and as a potential therapeutic target in, multiple myeloma. Targeted Oncol. (2018) 13:39–47. doi: 10.1007/s11523-017-0538-x, PMID: 29230672

[16] Shi F Xue R Zhou X Shen P Wang S Yang Y . Telitacicept as a BLyS/APRIL dual inhibitor for autoimmune disease. Immunopharmacol Immunotoxicol. (2021) 43:666–73. doi: 10.1080/08923973.2021.1973493, PMID: 34519594

[17] Wu D Li J Xu D Wang W Li L Fang J . A human recombinant fusion protein targeting B lymphocyte stimulator (BlyS) and a proliferation-inducing ligand (APRIL), telitacicept (RC18), in systemic lupus erythematosus (SLE): results of a phase 2b study. Arthritis Rheumatol. (2019) 71.

[18] Wu D Li J Xu D Wang L Fang J Ross D . Telitacicept, a human recombinant fusion protein targeting b lymphocyte stimulator (BlyS) and a proliferation-inducing ligand (APRIL), in systemic lupus erythematosus (SLE): results of a phase 3 study. Arthritis Rheumatol. (2022) 74:4546–8.

[19] Jin HZ Li Y Wang X Li Z Ma B Niu L . Efficacy and safety of telitacicept in patients with systemic lupus erythematosus: a multicentre, retrospective, real-world study. Lupus Sci Med. (2023) 10:e001074. doi: 10.1136/lupus-2023-001074, PMID: 38007228 PMC10679987

[20] Chen X Zhao Q Hou Y Jiang J Zhong W Wang W . Pharmacokinetics, pharmacodynamics, short term efficacy and safety of RCT-18, a novel BLyS/APRIL fusion protein, in patients with rheumatoid arthritis. Br J Clin Pharmacol. (2016) 82:41–52. doi: 10.1111/bcp.12908, PMID: 26917504 PMC4917793

[21] Zhao Q Chen X Hou Y Jiang J Zhong W Yao X . Pharmacokinetics, pharmacodynamics, safety, and clinical activity of multiple doses of RCT-18 in chinese patients with systemic lupus erythematosus. J Clin Pharmacol. (2016) 56:948–59. doi: 10.1002/jcph.686, PMID: 26634642

[22] Xie J Fan X Su Y Zhou H Cao S Zhu X . Pharmacokinetic characteristics, safety, and tolerability of telitacicept, an injectable recombinant human B-lymphocyte stimulating factor receptor-antibody fusion protein, in healthy chinese subjects. Clin Pharmacol Drug Dev. (2022) 11:1273–83. doi: 10.1002/cpdd.1136, PMID: 35844038 PMC9796261

[23] Ren Y Chen S Yang H . Case report: telitacicept in treating a patient with NF155+ autoimmune nodopathy: a successful attempt to manage recurrent elevated sero-anti-NF155 antibodies. Front Immunol. (2023) 14:1279808. doi: 10.3389/fimmu.2023.1279808, PMID: 37965304 PMC10642300

[24] Tian M Tang L . Good efficacy achieved by telitacicept, corticosteroids and immunosuppressants in the treatment of SLE combined with MOG-AD. Rheumatol Adv Pract. (2023) 7:rkad088. doi: 10.1093/rap/rkad088, PMID: 37937177 PMC10627278

